# Mercury, Lead, and Cadmium in the Muscles of Five Fish Species from the Mechraâ-Hammadi Dam in Morocco and Health Risks for Their Consumers

**DOI:** 10.1155/2021/8865869

**Published:** 2021-01-12

**Authors:** Mohammed Mahjoub, Soufiane Fadlaoui, Mohammed El Maadoudi, Youssef Smiri

**Affiliations:** ^1^Mohamed First University, Faculty of Sciences, Department of Biology, Laboratory of the Agricultural Production Improvement, Biotechnology, and Environment, P.B. 717, Oujda, Morocco; ^2^National Office of Food Safety (ONSSA), Regional Laboratory of Analysis and Research, P.B. 3, Tangier, Morocco

## Abstract

This study aims to assess the degree of metal contamination (mercury (Hg), cadmium (Cd), and lead (Pb)) in the muscles of five species of fish *Esox lucius*, *Sander lucioperca*, *Micropterus salmoides*, *Lepomis macrochirus*, and *Scardinius erythrophthalmus*, from the Mechraâ-Hammadi Dam between July 2017 and May 2018, and to conduct a risk assessment for human consumers. Trace metals were determined by Graphite Furnace Atomic Absorption Spectrometry for the Pb and the Cd and by Cold Vapor Atomic Absorption Spectrometry for the Hg. The results gotten from the study of the muscles of the different fish species show that the higher mean amounts of Cd and Hg were determined in *E. lucius*, and the maximum mean levels of Pb were detected in *S. erythrophthalmus*. Results suggested that demersal fishes inhabiting near the sediments and piscivorous fishes with higher trophic level were likely to accumulate higher trace metal concentrations. The general order of bioaccumulation of the trace metals measured in the muscles of the fish species is as follows: Hg > Pb > Cd. Therefore, the bioaccumulation of Hg in fish studied is more important than that of Cd and Pb. Furthermore, these concentrations are higher in summer than in winter for all trace metals. All the values of the trace metals in the muscle tissues are below the maximum limits recommended by the European Community (EC) N° 1881/2006. However, estimation of noncarcinogenic health risks by the target hazard quotient indicated no obvious noncarcinogenic risks to humans that consume those fishes (THQ < 1). Results of THQ and maximum safe consumption indicated that Hg may cause more harm to human by fish consumption especially for *E. lucius* and *S. lucioperca*. Therefore, reduced intake of carnivorous fishes should be promoted as part of a healthier diet.

## 1. Introduction

The aquatic ecosystems are continuously exposed to certain pollutants that are accompanied by increasing degradation of their quality [1]; this has a negative impact on the water quality and aquatic life of our ecosystems. The pollutants that could pose a threat to our aquatic ecosystems in general and fish fauna in particular are trace metals [2]. These elements are very dangerous because of their very high toxicity even at low concentrations, their prolonged persistence in the environment, and their tendencies to bioaccumulation in aquatic organisms because they are not biodegradable, and as a result, they concentrate large quantities in their tissues [3–5]. Trace metals reach aquatic environments, through the natural pollution due to soil erosion by rain, and by anthropogenic pollution due to liquid discharges results from different urban, industrial, and agriculture activities [6, 7]. In addition, revolving the bottom of the dam during the work of sludge removal may have made the metals available for the aquatic environment.

Located in Eastern Morocco, the Moulouya River is considered as the largest Moroccan river crossing several provinces from Midelt and leading to the Mediterranean. Three large dams are built on this river, including the Mechraâ-Hammadi Dam, which was commissioned in 1956 with a maximum storage capacity of around 6.6 million cubic meters [8]. It is a water reserve for irrigation and the production of drinking water; it also provides a fish-friendly habitat and an attractive tourist environment for sport and commercial fishing practitioners from the rural commune of Mechraâ-Hammadi and surrounding communities [9]. This dam is mainly powered by two rivers, the Moulouya River and the Za River which receive throughout their upstream courses, domestic and industrial liquid discharges loaded with trace metals are generated mainly by the cities of Taourirt and Guercif, and pollutants from agriculture in these areas [10, 11]. Therefore, the fish of Mechraâ-Hammadi Dam may find themselves exposed to high concentrations of trace metals that could have adverse effects on this fauna, from a quantitative and qualitative point of view, and become toxic for human consumption. Hence, knowing the values of trace metals in these fish is of great importance for the assessment of the potential health risks associated with the consumption of fish from the Mechraâ-Hammadi Dam.

There are no studies on the contamination of fish from the Mechraâ-Hammadi Dam, and it is in this context that we undertook this study, as part of assessing metal contamination, the most concern and most dangerous trace metals, Hg, Cd, and Pb, in the muscles of five species of fish Mechraâ-Hammadi Dam, in order to estimate the health risk they represent for their consumers by calculating the noncarcinogenic target hazard quotient (THQ) and maximum safe consumption (MSC) and to know the species most contaminated with trace metals. This study also introduces a spatiotemporal evaluation of the metallic pollution of the fish fauna of Mechraâ-Hammadi Dam.

## 2. Materials and Methods

### 2.1. Study Area

**Figure 1:**
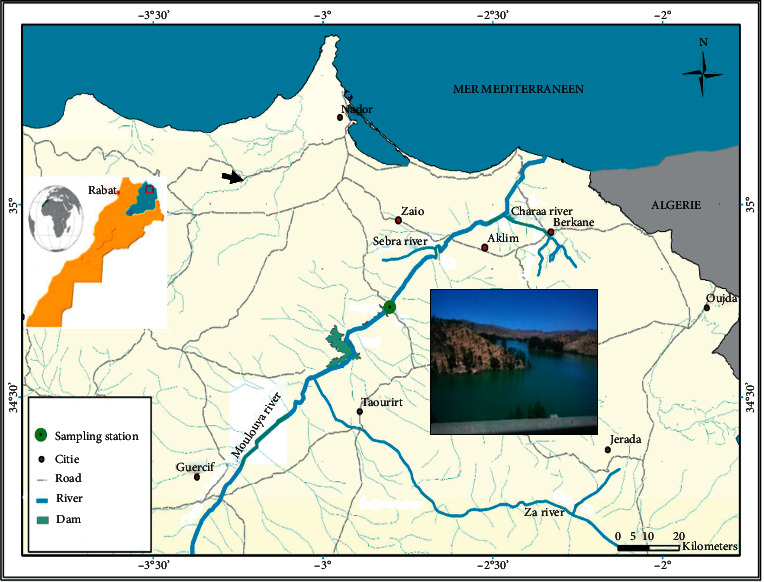
Localization map of the study area and location of sampling station area.

The Mechraâ-Hammadi Dam (34°44′05.0″ N 2°48′11.0″ W) is built on the Moulouya River, located 40 km far from the Mediterranean and 56 m above sea level in the province of Taourirt in the Eastern region of Morocco (Figure 1).

### 2.2. Sampling

Seasonal sampling missions were spread over the four seasons from summer of 2017 until the spring of 2018. The fish samples were captured with gill nets of various mesh sizes, after which they were euthanized by severing the spinal cord. We identified the species to which every fish belonged to using keys by Azeroual [12], and we put them in polyethylene bags, each bag of which contains individuals belong to the same species. The standard length (cm) and weight (g) were measured for every sample, using a vernier caliper and an analytical pocket balance, respectively, and they are transported in a cool box about 4°C to the laboratory, where they are stored in −25°C until the instant of the analysis.

### 2.3. Metal Analysis

In the Regional Laboratory of Analysis and Research of National Office of Food Safety (RLAR, ONSSA) in Tangier, the fish samples are thawed, headed, and eviscerated using stainless steel scalpels, and then we take the fish flesh (edible part), grounded, then homogenized in a domestic food blender. Subsequently, we pass on to the mineralization stage of the samples carried out according to the technique described by the AOAC Official Method [13]. Of which a quantity varying between 0.5 g and 0.6 g of wet weight sample is put in Teflon vessel in the presence of 5 ml of suprapur (69%) nitric acid (HNO_3_), for the samples that we evaluate the Hg content, and 5 ml of suprapur (69%) HNO_3_ and 2 ml of suprapur (30%) hydrogen peroxide (H_2_O_2_) for samples we assess Pb and Cd content. The Teflon vessels are hermetically closed and introduced into the microwave oven (Berghof speedway MWS-2) and gradually heated (for 45 min up to 185C°) until all the materials were dissolved. After digestion and cooling to room temperature, the samples are diluted by 50 ml with ultrapure water in polyethylene tubes. Pb and Cd are determined by Graphite Furnace Atomic Absorption Spectrometry (GF-AAS) (VARIAN PERKIN ELMER ACE 800), equipped with a fully automated autosampler system. 2.5% NH_4_H_2_PO_4_, and 1% Mg(NO_3_)_2_ are the applied matrix modifiers. Hg is quantified by Cold Vapor Atomic Absorption Spectrometry (CV-AAS) (VARIAN FIMS 100), in the presence of a reducing solution of stannous chloride (SnCl_2_) at 2.5%, and the carrier solution of hydrochloric acid (HCl) at 3%. For the two spectrometry, using high purity argon as the carrier gas, their flow rate is 50 ml/min.

All the tools used have been cleaned by soaking overnight in HNO_3_ (10%), rinsed with ultrapure water and dried, before each use. In addition to HNO_3_, the Teflon vessels have been cleaned with acetone. All of the reagents employed in this study are of analytical grade.

### 2.4. Quality Assurance and Quality Control

The calibration curve demonstrates a good linearity for the three trace metals, with correlation coefficients (*r*) greater than 0.999 (Table 1). On the other hand, the limit of detection (LD), the limit of quantification (LQ), the wavelength, and the standard calibration concentration of the present study are presented in Table 1.

According to ISO 17025 [14], the accreditation laboratories that perform analytical service must have quality control procedure for monitoring the validity of tests undertaken. The methods of GF-AAS and CV-AAS were accredited in the laboratory (RLAR, ONSSA), and the accuracy of the analytical methods was evaluated by participation to proficiency test schemes. The test materials distributed were canned fish at different concentrations of Cd, Pb, and Hg, obtained from the Food Analysis Performance Assessment Scheme (FAPAS). Replicate analysis of these proficiency tests showed good accuracy, with recovery rates for trace metals between 97.67% and 100.46% (Table 2).

### 2.5. Target Hazard Quotient (THQ)

The concentrations of trace metals in the muscle of the five fish species studied were used to assess the noncarcinogenic health risk from the consumption of this fish, using the formulas defined by the USEPA [15], and it has been reused by many authors [16–18] as follows:

(1) $$\begin{array}{c} {\text{EDI}}_{i}=\frac{C_{i}\times \text{DC}}{\text{BW}\times 1000},\\ {\text{THQ}}_{i}=\frac{{\text{EDI}}_{i}}{{\text{RfD}}_{i}},\\ \text{TTHQ}=\sum_{i}^{n}\text{THQ}, \end{array}$$

where EDI_*i*_ is the estimated daily intake of trace metal *i* in mg/kg/day. *C*_*i*_ is the mean concentration of trace metals *i* in the fish muscle in mg/kg. DC is the daily consumption rate of fish per person in g/day/person, estimated at 13.6 kg/year/person = 37.3 g/day/person for Morocco [19]. BW is the mean adult body weight of Moroccan adults and is estimated at 70.7 kg (72.1 kg for men and 69.3 kg for women) [20]. THQ_*i*_ is the target hazard quotient for trace metals *i*. RfD_*i*_ is the oral reference dose for trace metals *i* in mg/kg/day, and the RfD_*i*_ values of the trace metals studied are RfD_Hg_ = 3 × 10^−4^, RfD_Pb_ = 4 × 10^−3^, and RfD_Cd_ = 1 × 10^−3^ [16, 21]. TTHQ is the total target hazard quotient.

When THQ and TTHQ are <1, noncarcinogenic health risk is negligible, otherwise, it indicates greater risk and it is necessary to intervene and take protective action.

### 2.6. Maximum Safe Consumption (MSC)

To assess the maximum safe consumption (MSC) of the five fish species studied, using the provisional tolerable weekly intake (PTWI), the MSC was calculated according to the equation described by Metian et al. [22] as follows:

(2) $$\begin{array}{c} {\text{MSC}}_{i}=\frac{\text{BW}\times {\text{JL}}_{i}}{C_{i}}, \end{array}$$

where MSC_*i*_ is the maximum safe consumption of fooding/week items in relation with a contaminant *i*. BW is the body weight (kg) of the human for whom the assessment of the MSC_*i*_ is carried out. JL_*i*_ represents the PTWI of a trace metal *i*; according to the FAO/WHO [23], the PTWI for Cd, Pb, and Hg are 7, 25, and 4 *μ*g/kg/week, respectively. *C*_*i*_ is the mean concentration of a trace metal *i* in fish muscle in *μ*g/kg.

### 2.7. Statistical Analyses

Statistical analyses of the data were performed using the IBM SPSS Statistics 21 program. The level of statistical significance was defined at 95% (*p* < 0.05). A Pearson correlation test was used to examine the relationships between trace metals in the muscle tissue of different fish species.

## 3. Results

### 3.1. Trace Metals in Muscles of Fish Species

The number of fish and the biometric parameters (weight and length) of the fish species are summarized in Table 3. Also, the results of the evaluation of the mean concentrations of Hg, Pb, and Cd in the muscles of the different fish species collected from the Mechraâ-Hammadi Dam are shown in Table 4.

The results of Cd concentration in the analyzed fish species are low. They vary from 0.001 mg/kg of wet weight obtained in *L. macrochirus* to 0.005 mg/kg of wet weight in *E. lucius* (Table 4). An order of accumulation of Cd can be established in the muscles of the fish species studied. The result is obtained in Table 5, through which this order of bioaccumulation is slightly elevated in *E. lucius*.

The concentration of Pb revealed in the muscles of the fish studied shows that the lowest content is in the order of 0.017 mg/kg of wet weight in *M. salmoides*, while the highest value is in the order of 0.115 mg/kg of the wet weight obtained in *L. macrochirus* (Table 4). Pb accumulation order can be established, and the result is obtained in Table 5, of which this order of bioaccumulation is slightly elevated in *S. erythrophthalmus*.

For Hg concentrations measured in the muscles of fish, we have obtained results that vary from 0.056 mg/kg of wet weight in *M. salmoides* to 0.287 mg/kg of wet weight in *E. lucius* (Table 4). An order of accumulation of Hg can be established in the muscles of the fish species studied, and the result is obtained in Table 5. Of which this order of bioaccumulation is high in *E. lucius*.

Interelemental relationships in fish muscles (intermetal correlation) were assessed by the mean of Person's correlation coefficient, and this is presented in Table 6. No significant correlation was obtained at a level of significance (*p* < 0.05) which has been found between trace metals in different fish species of the Mechraâ-Hammadi Dam (Table 6).

Comparison of the mean trace metal concentrations detected in the muscles of the fish species studied shows that the Hg content are the highest compared to those of Pb and Cd, and the only variation is related to the high Pb content in *S. erythrophthalmus* (Table 7). From these results, we can establish the general order of bioaccumulation of the trace metals measured in the muscles of the different fish species which is as follows: Hg > Pb > Cd.

**Figure 2:**
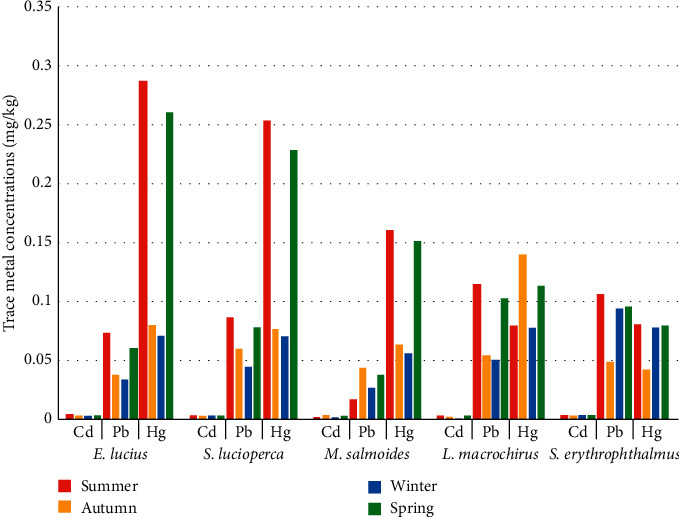
Seasonal variation in mean trace metal concentrations in muscles of the five fish species from the Mechraâ-Hammadi Dam in Morocco (mg/kg of wet weight).

Finally, for the seasonal variation in trace metals in the muscles of the fish species studied, important values are shown in summer, while winter records the lowest values in trace metals (Figure 2).

### 3.2. Estimation of Potential Public Health Risks

The estimation of the noncarcinogenic health risks to Moroccan people through the consumption of fish from the Mechraâ-Hammadi Dam was assessed by calculating the target hazard quotients (THQ_*i*_) and the total target hazard quotient (TTHQ). Results indicated that THQ values for individual trace metals varied among fishes, where the THQ results revealed that Hg had the highest values (0.1236 to 0.3069), followed by Pb (0.0041 to 0.0114), and then Cd (0.0012 to 0.0021) in this descending order Hg > Pb > Cd (Table 8). Whose THQ and TTHQ are <1 for all trace metals.

The maximum safe weekly consumption of fish species from the Mechraâ-Hammadi Dam has been calculated for adults who have the same average body weight of Moroccan adults (70.7 kg). The MSC for adult consumers was above 123,725 g/week for Cd, above 20,505 g/week for Pb, and above 1621 g/week for Hg (Table 9).

## 4. Discussion

Cd is a nonessential element and is considered one of the most toxic elements to humans, fishes, and environment, due to its capability of producing a chronic toxic effect even at a low concentration level [24]. These levels are all lower the European Community (regulation (EC) N° 1881/2006) for Cd in fish flesh, which is in the order of 0.05 mg/kg of wet weight for muscle meat of fish [25]. The lower concentration of Cd in muscle observed in this study suggests that muscle tissue is not an active site for the Cd accumulation process. In the scientific literature, similar values were recorded in the muscles of *L. macrochirus*, *B. callensis*, and *B. nasus* from the Moulouya River to Morocco [26], in the muscles of *L. graellsii*, *R. rutilus*, and *L. gibbosus* in the station 1 from the Llobergat River to Spain [27], and in *C. carpio* of Kasumigaura Lake in Japan [6]. While, low concentrations of Cd were found in the muscles of *B. barbus*, and *L. cephalus* from the Shahid Rajaei Dam in northern Iran [28], in the muscles of ten species caught at Šalek Lake in Slovenia [29], in fish *B. barbatula*, *S. cephalus*, and *B. barbus* from the Sûre River in Luxembourg [30], in three species from the Vaal Dam in South Africa [31], and in the muscles of *T. nilotica* from the High Dam Lake in Egypt [32]. On the other hand, some studies have shown high concentrations of Cd in the muscles of fish species, such as the study of Özparlak et al. [33] on nine fish species from Beyşehir Lake in Turkey, the work of Bahnasawy et al. [34] in the muscle of *M. cephalus* and *L. ramada*, and the study of Rajotte et al. [35] of the species *P. flavescens* in, namely, Ramsey, Nelson, Vermilion, and Whitson lakes in Canada (Table 10).

Similar to Cd, Pb is a serious environmental contaminant and is toxic to fish and human even in small quantities [24]. These levels detected in this study are all lower the European Community (regulation (EC) N° 1881/2006) for the Pb in fish flesh, which is in the order of 0.3 mg/kg of wet weight for muscle meat of fish [25]. The comparison with the data available in the literature indicated that similar concentrations of Pb were detected in the muscles of *L. kimberleyensis* from Vaal Dam to South Africa [36], in *L. macrochirus*, *B. callensis*, and *B. nasus* captured at the Moulouya River to Morocco [26], in the muscles of *L. graellsii*, *R. rutilus*, and *L. gibbosus* in the station 1 from the Llobergat River to Spain [27], and in *G. holbrooki* from Fouarat Lake and Sebou Estuary in Morocco [37]. However, low concentrations of Pb were found by Rashed et al. [32], Wariaghli et al. [38], Alam et al. [6], Boscher et al. [30], Plessl et al. [31], and Petkovšek et al. [29]. On the other hand, several studies have shown high concentrations of Pb, such as the study of Bahnasawy et al. [34] in the muscle of *M. cephalus* and *L. ramada*, the work of Özparlak et al. [33] on nine fish species, and the study of Alhas et al. [39] on *B. xanthopterus* and *B. rajanorum mystaceus* (Table 10). The order of bioaccumulation of Pb indicates that omnivorous fish living near sediments such as *S. erythrophthalmus* and *L. macrochirus* accumulated the largest quantities of this metal. This can be explained by the fact that these fish species are likely to have more contact with contaminated sediments, and as a result, metals contained in sediments are absorbed and stored in the fish tissues [27, 40]. This supported the hypothesis that the sediment was the major pathway for trace metal uptake for fish [41]. The high levels of Pb in *S. erythrophthalmus* and *L. macrochirus* compared to other species can also be attributed to the consumption of zoobentics, which have high concentrations of trace metals [42]. Our result agrees well with the conclusion of Yi et al. [42] on the order of accumulation of trace metals in different fish species, which is as follows: benthic invertivores > piscivores > zooplanktivores > phytophagic fishes > phytoplanktivores. Indeed, the origin of difference in bioaccumulation could be related to differences in diet, trophic level, metabolism, type of trace metal, preferred habitat, and lifestyle of these fish species [27, 43, 44].

Hg is a serious environmental pollutant and toxic to aquatic biota and humans even in low contents [5]. Hg amounts detected in this study are all below the European Community (regulation (EC) N° 1881/2006) for Hg in fish flesh, which is in the order of 0.5 mg/kg of wet weight in fish at the bottom of the food chain, and 1 mg/kg of wet weight in fish at higher in the food chain [25]. Comparing the results of our study with previous studies, we found that the study of Boscher et al. [30] presented similar results in fish *B. barbatula*, *S. cephalus*, and *B. barbus* captured at the Sûre River in Luxembourg, while this metal is not detected in the muscles of six species belong to the Atatürk Dam studied by Karadede et al. [45]. Other studies show low levels of Hg in fish muscles, such as the study of Petkovšek et al. [29] on ten species caught at Šalek Lake in Slovenia, the study of Mahjoub et al. [26] on *L. macrochirus*, *B. callensis*, and *B. nasus* captured at the Moulouya River in Morocco, the work of Mol et al. [46] on eight species of the Atatürk Dam in Turkey, and the study of Plessl et al. [31] on three species from the Vaal Dam in South Africa. On the contrary, some studies have shown high levels of Hg exceeding the recommended regulatory limits, such as the study of Dharampal et al. [47] on *M. salmoides* from the Sipsey River in the USA, the study of Scerbo et al. [48] on species of *A. anguilla*, *L. cephalus cabeda*, and *C. toxostoma* captured at the Cecina River in Italy, and the work of Shakeri et al. [28] on *B. barbus* from the Shahid Rajaei Dam in northern Iran (Table 10). We notice from the results of bioaccumulation order that the Hg concentrates in predatory fish are already recognized for their great power to accumulate trace metals, as seen in the species of *E. lucius*, *S. lucioperca*, and *M. salmoides* (mercury values up to 0.287; 0.254, 0.161 mg/kg of wet weight, respectively), and this fact can be explained by the affinity of Hg for the sulfhydryl groups of fish flesh proteins [49] and underlines that muscle is the main target of Hg storage [50]. Therefore, these relatively high concentrations of Hg in the muscles of these fish may not be directly related to their concentrations in water but are due to the phenomena of bioaccumulation and biomagnification [51]. The lowest contents of Hg were found in the muscle of *S. erythrophthalmus*, whose diet consists mainly of water plants, insects, and crustaceans [52].

The general order of metal bioaccumulation measured in the muscles of the different fish species studied was Hg > Pb > Cd. We have noted that Cd is the less accumulated metal, because he has a low tendency to accumulate in muscles [16, 53], where the concentrations are usually very low. Several studies have demonstrated that Cd preferentially accumulates in active metabolic organs, such as kidney and liver [16]. Unlike Cd, Hg preferentially accumulates in muscles due to their affinity for the sulfhydryl groups of proteins [49]. Therefore, there is an evidence for Hg bioaccumulation and biomagnification; however, evidence for Cd biomagnifications is inconsistent [2].

The fish of the Mechraâ-Hammadi Dam has relatively high values in trace metals, and particularly for Hg and Pb, this station is permanently subject to the urban contributions of the agglomerations located upstream of the Mechraâ-Hammadi Dam like that of Taourirt and Guercif, as well as the discharges of the oil mills of Taourirt and Guercif [8, 10]. These discharges can be considered as the most important sources of pollution of fish by trace metals studied. Additionally, the possibility of leaching of agricultural lands of the surroundings areas of this reservoir can be given in terms of sources of trace metals in this station.

Seasonal variations were also observed in the data collected. Higher values were obtained during summer season than during winter season, and this may be due to the increase in the volume of water in the dam by the supply of storm water, induced their dilutions in winter. Moreover, the very high temperatures in the summer could also lead to higher metabolic rates, which could induce an increase in fish food activity, and this in turn increases the concentration of metals in fish [26, 55, 56]. This result agrees well with some studies [26, 56, 57].

Concerning the estimation of the potential public health risks, noncarcinogenic health risk was assessed by the calculation of THQ and TTHQ values for Cd, Pb, and Hg from the consumption of fish by adults. In the present study, THQ and TTHQ results were fewer than 1 in adult consumers for all three trace metals, suggesting that people would not experience significant health risks from the intake of fish species from the Mechraâ-Hammadi Dam. However, according to the calculation formula already cited, the value of THQ depends on the trace metal concentration in fish, the age and weight of consumers, and the rate of fish ingestion. Therefore, excess fish consumption in a Mechraâ-Hammadi Dam population may easily to THQ > 1. In this study, the major risk contributor is Hg, with the highest THQ value of 0.3069 and 0.2775 for *E. lucius* and *S. lucioperca*, respectively, in adult consumers.

For the maximum safe weekly consumption, Hg appears as the only trace metal of concern regarding the consumption of Mechraâ-Hammadi Dam fish (mainly for predatory fish: *E. lucius and S. lucioperca*), where the maximum amount of *E. lucius* and *S. lucioperca* that should be eaten by a 70.7 kg adult person to reach the PTWI for Hg is about 1621 g and 1792 g over a week, respectively. These results indicated that Hg may cause more harm to human by fish consumption, and the consumption of *E. lucius* and *S. lucioperca* should be limited the most (should be below 1621 g/week and 1792 g/week, respectively) in order to avoid the negative effect of Hg, while the concentrations of Cd and Pb in fish species were safe for consumption.

## 5. Conclusions

The results obtained after the dosage of Hg, Pb, and Cd in the muscles of five fish species captured from the Mechraâ-Hammadi Dam allow us to conclude as follows:

- Hg contents are the highest compared to those of Pb and Cd, whose general order of metal bioaccumulation measured in the muscles of the different fish species is Hg > Pb > Cd.
- The accumulation of metals was more pronounced in *E. lucius* (carnivorous fish) for Hg and Cd and was more pronounced in *S. erythrophthalmus* (demersal fish) for Pb. Metal levels vary between fish species due to their differences in food habits, type of trace element, and their lifestyles.
- Summer is the season during which it records the highest concentrations of metals in the fish studied while winter records the lowest concentrations.
- The concentrations of the three metals in the muscles of the fish studied have values below the regulatory limits of the European Community (EC). According to the THQ and MSC calculations, Hg may cause nonignorable health effects in humans if these species (especially *E. lucius* and *S. lucioperca*) are consumed at a larger amount, whereas remaining elements will not pose any adverse health effects to humans.

## Figures and Tables

**Table 1:** The wavelength, limit of detection (LD), limit of quantification (LQ), standard calibration concentration, and correlation coefficient (*r*) for trace metal determination.

Trace metals	Wavelength (nm)	LD (*μ*g/kg)	LQ (*μ*g/kg)	Standard calibration concentration (*μ*g/l)	Correlation coefficient (*r*)
Cd	228.8	0.0027	0.01	0, 0.1, 0.2, 0.4, 0.8, 1.6, 3.2	0.999830
Pb	283.3	0.76	2.5	0, 1, 2, 4, 8, 16, 32	0.999791
Hg	253.7	0.2	0.4	0, 1, 2.5, 5, 10, 20	0.999902

**Table 2:** Trace metal determination in proficiency tests (FAPAS canned fish samples).

Trace metals	Reference value (mg/kg) ± SD	Observed value (mg/kg) ± SD	Recovery (%)
Cd	6.2100 ± 1.5100	6.0655 ± 0.2636	97.67
Pb	0.0526 ± 0.0232	0.0515 ± 0.0083	97.91
Hg	0.1080 ± 0.0455	0.1085 ± 0.0129	100.46

SD: standard deviation.

**Table 3:** Number of fish species and weight and length of the studied fish species.

Species	*n*	Weight (g)		Length (cm)	
		$\bar{X}$ ± SD	*m*–*M*	$\bar{X}$ ± SD	*m*–*M*
*E. lucius*	10	451.7 ± 231.98	303–1095	40.75 ± 6.86	35.1–58.6
*S. lucioperca*	9	427.89 ± 94.98	283–610	37.49 ± 2.99	33.1–42.6
*M. salmoides*	23	148.13 ± 48.52	51–215	21.7 ± 2.57	15.5–25.2
*L. macrochirus*	35	75.83 ± 23.16	35–130	14.5 ± 2.78	9.3–20.1
*S. erythrophthalmus*	22	372.24 ± 91.35	210–580	25.93 ± 4.96	17–36

*n*: number of fish; $\bar{X}$: mean; SD: standard deviation; *m*: minimum; *M*: maximum.

**Table 4:** Contents of trace metals dosed in the muscles of fish species collected from the Mechraâ-Hammadi Dam (mg/kg of wet weight).

Species	Cd		Pb		Hg	
	$\bar{X}$ ± SD	*m*–*M*	$\bar{X}$ ± SD	*m*–*M*	$\bar{X}$ ± SD	*m*–*M*
*E. lucius*	0.004 ± 0.0008	0.003–0.005	0.0518 ± 0.019	0.034–0.074	0.1745 ± 0.1149	0.071–0.287
*S. lucioperca*	0.0033 ± 0.0005	0.003–0.004	0.0675 ± 0.0187	0.045–0.087	0.1578 ± 0.0973	0.071–0.254
*M. salmoides*	0.0028 ± 0.0009	0.002–0.004	0.0313 ± 0.0116	0.017–0.043	0.108 ± 0.0557	0.056–0.161
*L. macrochirus*	0.0023 ± 0.0009	0.001–0.003	0.0810 ± 0.0327	0.051–0.115	0.1028 ± 0.0296	0.078–0.140
*S. erythrophthalmus*	0.0038 ± 0.0005	0.003–0.004	0.0862 ± 0.0254	0.049–0.106	0.0703 ± 0.0182	0.043–0.081

$\bar{X}$: mean; SD: standard deviance; *m*: minimum, *M*: maximum.

**Table 5:** The order of the bioaccumulation of trace metals in the muscles of the studied fish species.

Trace metals	Order of metal bioaccumulation
Cd	*E. lucius* > *S. erythrophthalmus* > *S. lucioperca* > *M. salmoides* > *L. macrochirus*
Pb	*S. erythrophthalmus* > *L. macrochirus* > *S. lucioperca* > *E. lucius* > *M. salmoides*
Hg	*E. lucius* > *S. lucioperca* > *M. salmoides* > *L. macrochirus* > *S. erythrophthalmus*

**Table 6:** Matrix of correlation between the different trace metals in different fish species.

	Cd	Pb	Hg
Cd	1		
Pb	0.16742944	1	
Hg	0.26232459	−0.4331575	1

**Table 7:** Order of trace metals accumulated in the fish muscles from the Mechraâ-Hammadi Dam.

Species	Order
*E. lucius*	Hg > Pb > Cd
*S. lucioperca*	Hg > Pb > Cd
*M. salmoides*	Hg > Pb > Cd
*L. macrochirus*	Hg > Pb > Cd
*S. erythrophthalmus*	Pb > Hg > Cd

**Table 8:** Target hazard quotient (THQ) and total THQ (TTQH) calculated for each species.

Species	THQ			TTHQ
	Cd	Pb	Hg	
*E. lucius*	0.0021	0.0068	0.3069	0.3158
*S. lucioperca*	0.0017	0.0089	0.2775	0.2882
*M. salmoides*	0.0015	0.0041	0.1899	0.1955
*L. macrochirus*	0.0012	0.0107	0.1808	0.1927
*S. erythrophthalmus*	0.0020	0.0114	0.1236	0.1370

**Table 9:** Maximum safe consumption for Cd, Pb, and Hg in fish from the Mechraâ-Hammadi Dam (g fish/week).

Species	Cd	Pb	Hg
*E. lucius*	123725	34122	1621
*S. lucioperca*	149970	26185	1792
*M. salmoides*	176750	56470	2619
*L. macrochirus*	215174	21821	2751
*S. erythrophthalmus*	130237	20505	4023

**Table 10:** Comparisons of recorded trace metal levels in the muscles of the fish species during the present study with the literature reported from different areas (mg/kg of wet weight).

Fish species	Region	Cd	Pb	Hg	References
*C. carpio*	Kasumigaura Lake (Japan)	0.009	0.031	—	[6]
*L. macrochirus*, *B. callensis*, and *B. nasus*	Moulouya River (Morocco)	0.001–0.006	0.016–0.200	0.009–0.187	[26]
*L. graellsii, R. rutilus*, and *L. gibbosus*	Station 1 from the Llobregat River (Spain)	0.007, 0.004, 0.009, resp.	0.095, 0.107, 0.078, resp.	—	[27]
*B. barbus* and *L. cephalus*	Shahid Rajaei Dam (Iran)	0.018, 0.04, resp.	—	0.7, 0.37, resp.	[28]
*A. brama danubii, A. alburnus, B. meridionalis petenyi, C. auratus gibelio, C. carpio, L. gibbosus, L. cephalus, P. fluviatilis, R. rutilus,* and *S. erythrophthalmus*	Šalek Lake (Slovenia)	≤0.01	0.01–0.04	0.03–0.16	[29]
*B. barbatula, S. cephalus*, and *B. barbus*	Sure River (Luxembourg)	0.024, 0.027, 0.028, resp.	0.037, 0.018, 0.034, resp.	0.037, 0.298, 0.096, resp.	[30]
*L. aeneus, L. kimberleyensis,* and *L. umbratus*	Vaal Dam (South Africa)	<0.0012	0.012, 0.007, 0.008, resp.	0.096, 0.133, 0.05, resp.	[31]
T. nilotica	High Dam Lake (Egypt)	0.018	0.13	—	[32]
*P. anatolicus, A. akili, G. gobio microlepidotus, L. lepidus, C. gibelio, C. carpio, S. erythrophthalmus, S. lucioperca*, and *T. tinca*	Beyşehir Lake (Turkey)	0.8, 0.8, 0.47, 0.43, 0.46, 0.43, 0.46, 0.43, 0.55, resp.	0.57, 1.05, 0.71, 0.4, 0.5, 0.57, 0.39, 0.32, 0.67, resp.	—	[33]
*M. cephalus and L. ramada*	Manzala Lake (Egypt)	0.216–0.334, 0.102–0.222, resp.	0.332–0.596, 0.286–0.486, resp.	—	[34]
*P. flavescens*	A range of Lakes (Canada)	0.338–2.598	—	—	[35]
*L. kimberleyensis*	Vaal Dam (South Africa)	—	0.101	—	[36]
*G. holbrooki*	Fouarat Lake and Sebou Estuary (Morocco)	—	0.0002–0.1967	—	[37]
*A. anguilla*	Moulay Bousselham Lagoon (Morocco)	—	0.002	—	[38]
*B. xanthopterus,* and *B. rajanorum mystaceus*	Atatürk Dam (Turkey)	Not detected	0.68, 0.66, resp.	—	[39]
*C. regium, A. marmid, C. trutta, C. mossulensis, C. luteus,* and *C. carpio*	Atatürk Dam (Turkey)	Not detected	Not detected	Not detected	[45]
*S. triostegus, A. marmid, A. vorax, C. trutta, C. luteus, C. mossulens*, and *C. carpio*	Atatürk Dam (Turkey)	—	—	0.011–0.130	[46]
*M. salmoides*	Sipsey River (USA)	—	—	0.87	[47]
*A. anguilla, L. cephalus cabeda*, and *C. toxostoma*	Cecina River (Italy)	—	—	0.82, 0.558, 0.65, resp.	[48]

Tissue concentrations found in dry weight were converted to wet weight by multiplying by a factor of 0.2 (considering an average water content in fish tissues of 80%) [54].

## Data Availability

The data used to support the findings of this study are available from the corresponding author upon request.
